# MiR-30a regulates cancer cell response to chemotherapy through SNAI1/IRS1/AKT pathway

**DOI:** 10.1038/s41419-019-1326-6

**Published:** 2019-02-15

**Authors:** Tingting Wang, Gang Chen, Xuemei Ma, Yao Yang, Yali Chen, Yihan Peng, Zhigang Bai, Zhongtao Zhang, Huadong Pei, Wei Guo

**Affiliations:** 1 Department of General Surgery, Beijing Friendship Hospital, Capital Medical University, 100050 Beijing, China; 2 State Key Laboratory of Proteomics, National Center for Protein Sciences Beijing, Beijing Proteome Research Center, Beijing Institute of Lifeomics, 102206 Beijing, China; 3 National Clinical Research Center of Digestive Diseases, 100050 Beijing, China; 4 Department of Integrated Traditional Chinese Medicine and Western Medicine, Tongji Hospital, Huazhong University of Science and Technology, 430030 Wuhan, China; 5 George Washington University Cancer Center, USA; Department of Biochemistry and Molecular Medicine, The George Washington University School of Medicine and Health Science, 2300 Eye Street, N.W, Washington, DC 20037 USA

## Abstract

Despite gemcitabine being the leading chemotherapeutic drug for pancreatic cancer, many patients still relapse due to the drug resistance. We previously reported the molecular link between FKBP51 mediated AKT inhibition and gemcitabine response in pancreatic cancers. However, the upstream regulator of this pathway, especially the involvement of non-coding RNAs in gemcitabine response is still not clear. Here we delineated the miRNA expression profile and key signaling pathways associated with gemcitabine response. Furthermore, we confirmed that miR-30a, one node of this network, regulated cellular response to gemcitabine through SNAI1-IRS1-AKT pathway. MiR-30a directly targeted SNAI1, which activates AKT and ERK through regulating IRS1 in vitro and in vivo. Clinically, miR-30a is downregulated in pancreatic cancer tissue and associated with overall patient survival. We also identified miR-30a as an AKT-FOXO3a-regulated gene that forms a feedback loop. Together, these results demonstrate that miR-30a is an upstream regulator of the Akt pathway with a critical role in cancer etiology and chemoresistance.

## Introduction

Pancreatic adenocarcinoma (PDAC) is one of the most lethal malignant tumor type in the world, displaying 5-year overall survival rate of no more than 7%^1^. Due to the locally invasive and metastatic nature of this disease, adjuvant chemotherapy is the core treatment option for 80% of the pancreatic cancer patients, as surgical operation cannot be performed^2^. Among different chemotherapeutic agents, gemcitabine (GEM) has been a gold-standard for the treatment of advanced pancreatic cancer patients, and has been observed to effectively improve the patient prognosis^3,4^. Nonetheless, in the majority of patients, resistance to GEM inevitably develops, leading to treatment failure^5^. Therefore, understanding resistance mechanisms and expanding the therapeutic utility of GEM will improve patient’s prognosis significantly.

AKT pathway is fundamental in mediating multiple cellular processes, including cell proliferation and survival^6^, angiogenesis^7^, and glucose metabolism^8^. AKT hyperactivation has been shown to be associated with cancer predisposition and chemoresistance^9,10^. AKT is also one of the most commonly upregulated oncogene in multiple cancers. Therefore, due to the central signaling node status of AKT within the cells, its activity needs to be strictly regulated. We previously reported that the scaffolding protein, immunophilin FKBP51 enhances PHLPP–AKT interaction, and facilitates PHLPP-mediated AKT dephosphorylation at Ser473 residue. FKBP51 affects AKT activation and gemcitabine resistance in pancreatic cancer cells^11^. More recently, we found that SIRT7 interacted with FKBP51, and deacetylated FKBP51 at lysines 28 and 155 residues (K28 and K155), thereby resulting in enhanced interactions among FKBP51, AKT, and PHLPP, and culminated in AKT dephosphorylation and subsequent sensitization of cancer cells against gemcitabine^12^. However, the upstream regulator of this pathway, especially the involvement of non-coding RNAs in AKT activation and gemcitabine response is still not clear.

MicroRNAs (miRNAs), which are small RNAs usually 19–23 bp in length or shorter, have been implicated in regulating the expression and function of protein-coding RNAs. Aberrant levels of miRNA have been reported in variety of human cancers^13–15^. There has been a strong evidence regarding involvement of miRNAs in tumor growth^16–18^, invasion^19^, angiogenesis^20,21^, and immune evasion^22^, through targeting specific mRNAs, thereby reinforcing the notion about their importance in regulation of overall cellular functions. Recently, the functions of miRNAs in drug resistance have also started to emerge^23–25^. However, miRNAs functions in pancreatic cancer etiology and chemo-response are still not fully understood. In this study, we delineated a genome wide miRNAs expression profile and identified key pathways associated with gemcitabine response in pancreatic cancer cells. Furthermore, we validated several miRNAs nodes that have been dysregulated during gemcitabine resistance, and subsequently confirmed the role of miR-30a in pancreatic cancer cell sensitization to chemotherapy, mainly through SNAI1/IRS1/ERK/AKT pathway.

## Results

### Deep sequencing of small RNAs associated with gemcitabine response

**Fig. 1:**
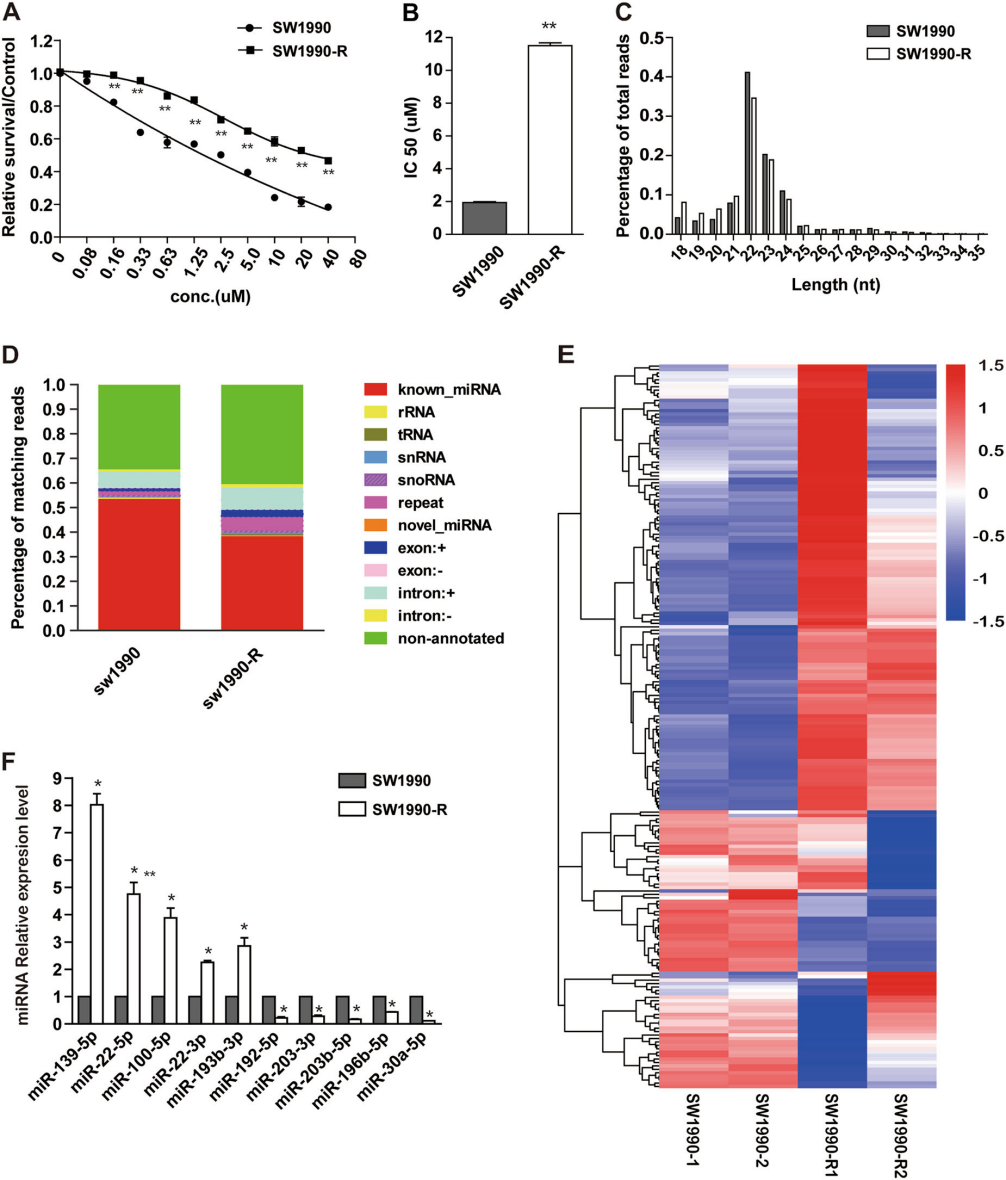
Deep sequencing of small RNAs associated with gemcitabine response. **a**, **b** Drug-resistance cell line was established as described in the Materials and methods. Gemcitabine sensitivity of both SW1990-R cells and its parental cells (**a**) were tested by MTS assay and the IC50 values were calculated (**b**). *P < 0.05 and **P < 0.01, compared with control conditions. **c** Sequence length distribution in SW1990-R and SW1990 cells. **d** Summary of sequencing reads mapped to various types of sRNA in SW1990-R and SW1990 cells. **e** Cluster analysis of changed miRNAs in SW1990-R cells compared with SW1990 cells. A line indicates a microRNA gene, and a column indicates a sample. Two samples were contained in each group. Both upregulated (red) and downregulated miRNAs (blue) were identified. **f** Expression**s** of ten identified miRNAs were detected by qRT-PCR to validate microarray data. *P < 0.05, compared with SW1990. Points, mean values for three independent experiments; error bars, ±SEM

To determine chemo sensitivity of pancreatic cancer cells to gemcitabine, five different cell lines were treated with gemcitabine at different concentrations for 72 h, cell viability was examined by MTS assay and 50% inhibition concentration (IC50) was calculated. As shown in Supplementary Fig. 1A and 1B, the IC50 of SW990, BxPC-3 are much lower than PANC-1 and Mia-PaCa-2 cells. To establish gemcitabine-resistant pancreatic cancer cells, SW1990 cells were selected and treated with gemcitabine, in a continuous stepwise fashion. Subsequent cell viability analyses revealed that the IC50 of SW1990-R (11.51 μM) increased by six fold compared with the parental SW1990 cells (Fig. 1a, b).

Next, to identify dysregulated small RNAs in SW1990-R cells, we performed a high-throughput RNA sequencing analysis. It generated 17.52 and 17.09 million (M) raw reads in SW1990-R cells and SW1990 parental cells, respectively. Among these, 17.13 and 16.38 M reads were selected as high-quality data, after processing and filtering criteria. In addition, length distribution assay indicated wide variation, ranging from 18 to 35 nucleotides (nt), but 22 nt small RNAs were the most abundant (Fig. 1c). These reads were regarded as reliable miRNA candidates for subsequent analysis. 87.94% and 93.22% high-quality reads from SW1990-R and SW1990 cells, respectively, were aligned with human genome (release hg19). As shown in Fig. 1d, around 40 to 50% of the mapped small RNAs accounted for miRNAs annotated in miRBase (release 20.0). However, other small RNA species, like small nuclear RNAs (snRNAs) and small nucleolar RNAs (snoRNAs) were also identified, but with lower abundance. Interestingly, the 34.5 to 40.5% of the mapped reads corresponded with non-annotated small RNAs (Fig. 1d). Overall, the complete set of detected mature miRNAs has been shown in Additional file 1.

### Identification of differentially expressed miRNAs associated with gemcitabine response

Next, we analyzed the miRNAs expression profile difference between gemcitabine-resistant and sensitive SW1990 cells. The expression value of each miRNA was normalized using transcripts per million (TPM) in each sRNA library. As shown in Fig. 1e, 136 miRNAs displayed differential expression between these two groups, based on *P*-value of below 0.05. Among these 61 miRNAs were upregulated, while 75 were downregulated in SW1990-R cells, in comparison to parental SW1990 cells, thereby suggesting that perhaps miRNAs are linked to gemcitabine response. We confirmed 10 miRNAs expression change using qRT-PCR (Fig. 1f), and observed the consistency between the results obtained from deep sequencing and from qRT-PCR, thus establishing the credibility of the data.

### Prediction of gemcitabine responsive miRNAs

To predict the target genes of miRNAs, we used the miRanda algorithm^26^ (http://www.microrna.org). 6642 potential target genes were identified against 136 miRNAs. Based on the predicted genes, we performed gene ontology (GO) and pathway analysis to obtain information about the significant biological functions and pathways involved in gemcitabine response.

**Fig. 2:**
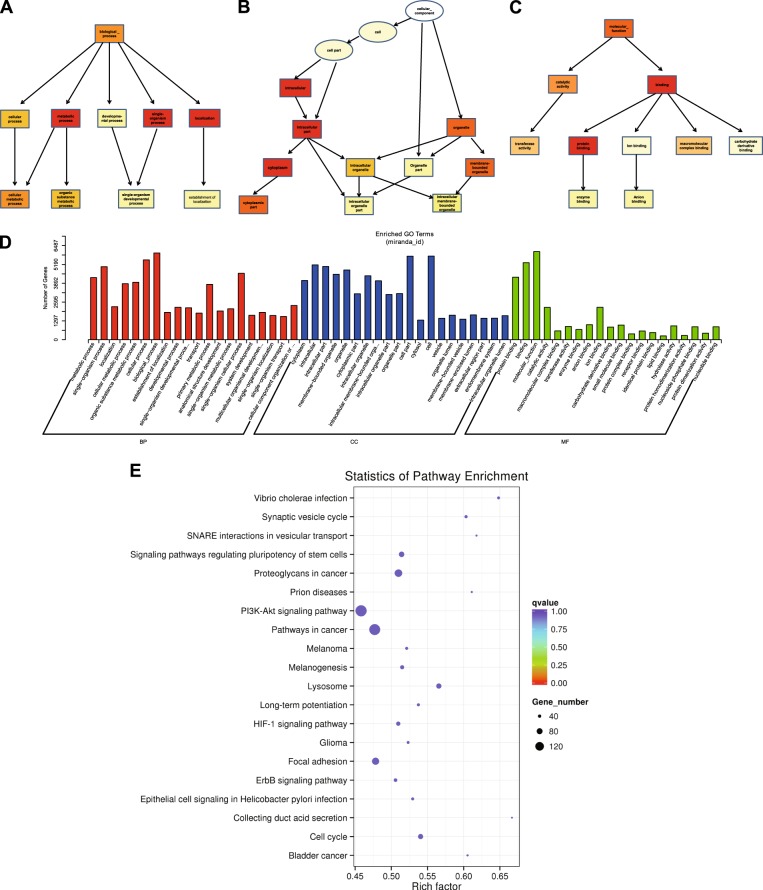
GO and KEGG pathway analysis of dysregulated miRNA-mRNAs. **a**–**d** GO analysis of dysregulated miRNA-mRNAs between SW1990-R and SW1990 cells. The significantly enriched biological process (**a**), cellular component (**b**), and molecular function (**c**) were showed by Directed Acyclic Graph (DAG). GO terms in box are enriched ones while those in circle are non-enriched parents. Color depth represents the enrichment degree. The number of dysregulated genes in each significantly enriched GO term was displayed as histogram (**d**). **e** Analysis of top 20 overrepresented KEGG pathways between SW1990-R and SW1990 cell line. The size of each circle stands for the number of dysregulated genes enriched in corresponding pathway. The rich factor was calculated using the number of enriched genes divided by the number of all background genes in corresponding pathway. *Q*-value was calculated using the Benjamini–Hochberg correction. When the number of genes is greater, the rich factor is greater and the *Q*-value is closer to zero, then the enrichment is more significant

The GO enrichment results have been depicted as graphical presentation using directed acyclic graph (DAG), where each branch represents relationship of inclusion, and defines the function scope, that gradually reduce from top to bottom. The top 10 results of GO enrichment analysis were selected as the master node of DAG and showed together with related GO term via inclusion relationship. The DAG representing biological process (BP), cellular component (CC) and molecular function (MF) have been shown in Fig. 2a–c. On the other hand, the genes involved in significantly enriched GO term were statistically analyzed and displayed as histogram (Fig. 2d).

Moreover, we also performed KEGG pathway enrichment analysis and has been displayed in an enriched scatter diagram of the predicted target genes, where the degree of KEGG enrichment was evaluated by *Q*-value, Rich factor and number of genes. As observed in Fig. 2e, “PI3K-Akt signaling pathway” and “Pathways in cancer” seems to be the major pathways involved in gemcitabine response.

Among the differentially expressed miRNAs, miR-30a showed obvious downregulation in SW1990-R cells. More importantly, its targets are involved in gemcitabine response, including IRS1, which is an upstream regulator of AKT, the pathway highlighted in KEGG enrichment analysis (Fig. 2e). Therefore, we are interested in exploring the possible roles of miR-30a in gemcitabine response.

### MiR-30a overexpression suppresses cell proliferation, and sensitizes pancreatic cancer cells to gemcitabine

**Fig. 3:**
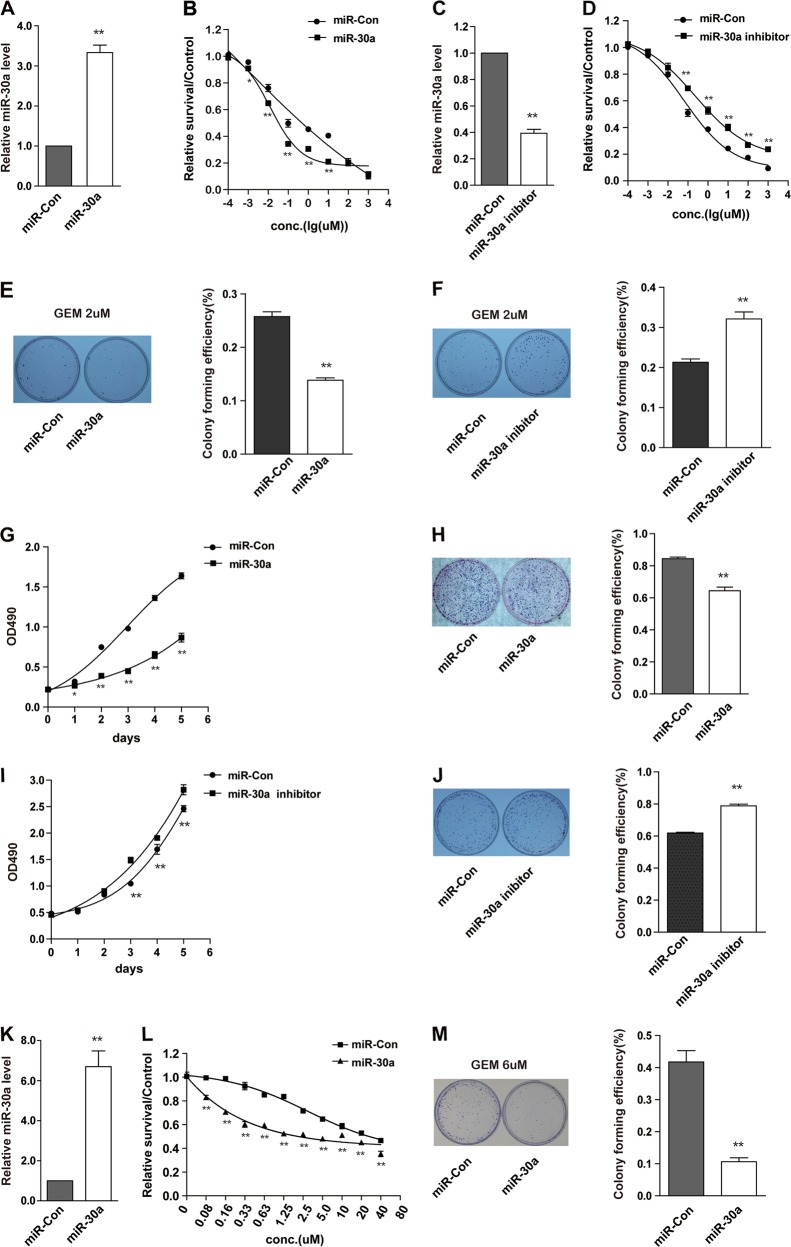
MiR-30a suppresses cell proliferation and enhances cell sensitivity to gemcitabine. **a**, **b** SW1990 cells were transfected with miR-30a precursor (miR-30a) or scramble control lentivirus (miR-Con). MiR-30a level was confirmed by qRT-PCR (**a**). Gemcitabine sensitivity was determined by MTS assays (**b**). *P < 0.05 and **P < 0.01, compared with miR-Con. **c**, **d** SW1990 cells were transfected with miR-30a inhibitor or scramble control (miR-Con). MiR-30a expression was detected by qRT-PCR (**c**). Gemcitabine sensitivity was determined by MTS assays (**d**). *P < 0.05 and **P < 0.01, compared with miR-Con. **e** SW1990 cells were transfected with indicated constructs and then treated with 2 μM gemcitabine for 72 h. The colony forming assay was performed. Representative micrographs (left) and quantification (right) of crystal violet-stained cell colonies were displayed. **f** SW1990 cells were transfected with indicated constructs. Cells were then treated and detected as in **e**. **g**, **h** SW1990 cells were transfected with miR-30a or miR-Con. Cell growth were determined by MTS assays (**g**). Representative micrographs (left) of colony formation, as well as quantification (right) of crystal violet-stained cell colonies were displayed (**h**). *n* = 3 wells per group. *P < 0.05 and **P < 0.01, compared with miR-Con. **i**, **j** SW1990 cells were transfected with miR-30a inhibitor or miR-Con. Cell growth and colony formation assay were determined as in **g** and **h**, respectively. *n* = 3 wells per group. *P < 0.05 and **P < 0.01, compared with miR-Con. **k**, **l** SW1990-R cells were transfected with miR-30a precursor (miR-30a) or scramble control lentivirus (miR-Con). MiR-30a level was confirmed by qRT-PCR (**k**). Gemcitabine sensitivity was determined by MTS assays (**l**). *P < 0.05 and **P < 0.01, compared with miR-Con. **m** SW1990-R cells after transfection with indicated constructs were treated with 6uM gemcitabine for 72 h. The colony forming assay was performed as in **e**. Points, mean values for three independent experiments; error bars, ±SEM

To verify the relationship between miR-30a and chemoresistance, we analyzed miR-30a expression levels in five pancreatic cancer cell lines. As shown in Supplementary Figure 1C, miR-30a level is negatively correlated with drug resistance. To further investigate the roles of miR-30a in pancreatic cell response to gemcitabine, we overexpressed or inhibited miR-30a in pancreatic cancer cells. As shown in Fig. 3a and b, miR-30a overexpression in SW1990 cells sensitized them to gemcitabine. Consistent with this result, inhibition of miR-30a increased gemcitabine resistance in SW1990 cells (Fig. 3c, d). Similar results were also observed in Capan-2 cells (Supplementary Fig. 2A–D). In addition, to elucidate the underlying mechanism, we performed the MTS and colony formation assay. As shown in Fig. 3e–j, overexpression of miR-30a inhibited colony formation and cell proliferation in SW1990 cells with or without 2 μM gemcitabine treatment, while inhibition of miR-30a promoted cell proliferation and colony formation. When we overexpressed or knocked down miR-30a in Capan-2 cells (Supplementary Figure 2A and 2C), we got the similar results (Supplementary Figure 2E–J). Furthermore, overexpression of miR-30a in SW1990-R cells partly restored its sensitivity to gemcitabine (Fig. 3k–m). Based on above results, we concluded that miR-30a has a critical role in pancreatic cancer cell growth and response to gemcitabine.

### MiR-30a functions through SNAI1/IRS1/AKT/ERK pathway

**Fig. 4:**
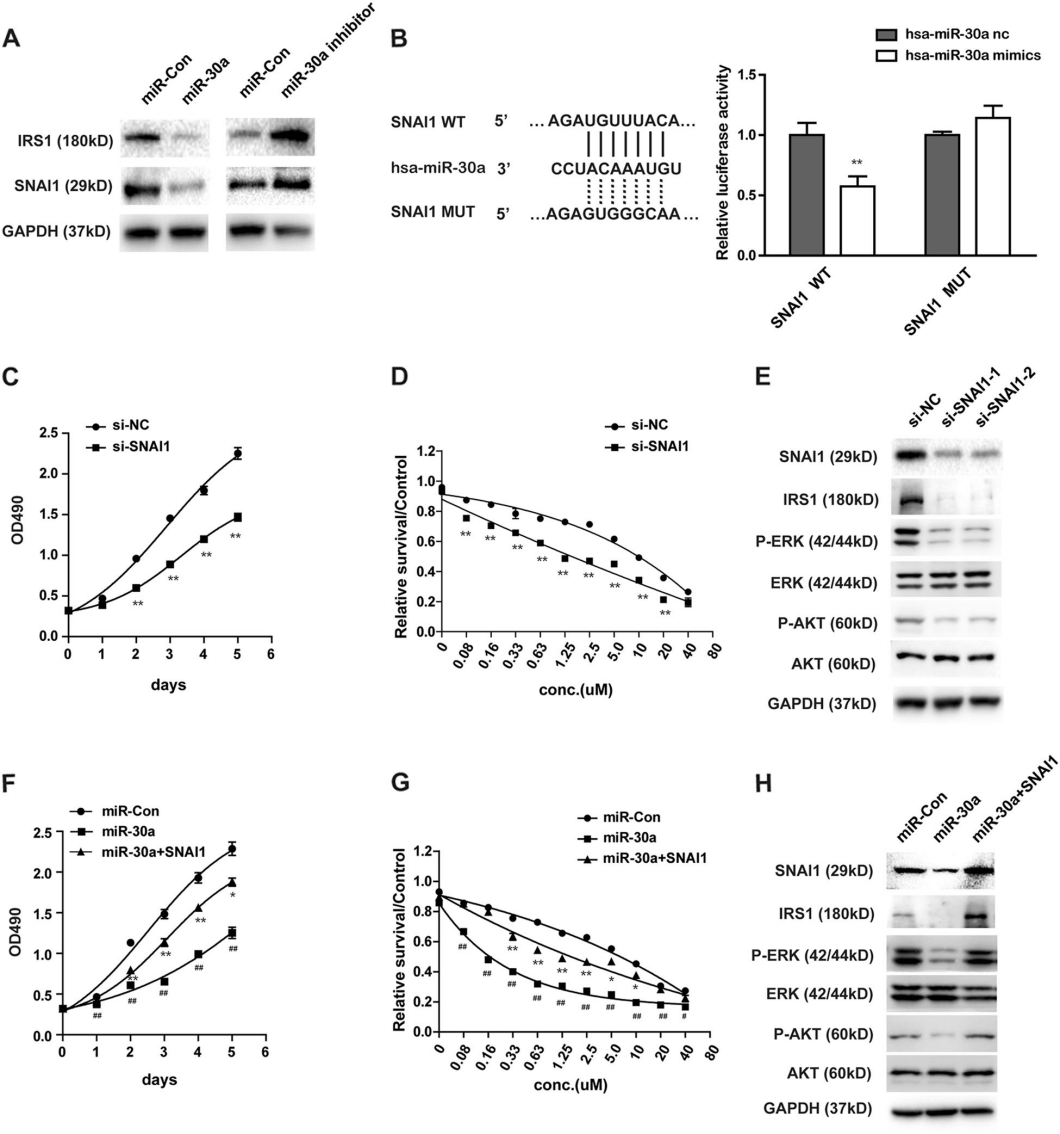
MiR-30a functions as tumor suppressor partly through SNAI1/IRS1/AKT/ERK pathway. **a** The effect of miR-30a modulation (miR-con, miR-30a and miR-30a inhibitor) on IRS1 and SNAI1 protein expression by western blot analysis in SW1990 cells. **b** MiR-30a targets SNAI1 directly. Representative seed sequences for miR-30a on the SNAI1 3′UTR was shown (left). 293T cells were transiently transfected with pmirGlo plasmids encoding wild-type or mutated 3′UTR sequences of SNAI1, and oligos. Luciferase activities were then measured 24 h after transfection (right). Firefly luciferase was normalized to Renilla luciferase. The data are represented as mean ± SEM (*n* = 3). **c**–**e** Knockdown of SNAI1 by transfection of two different SNAI1 siRNAs (si-SNAI1–1, si -SNAI1–2) in SW1990 cells. Cell growth (**c**) and gemcitabine sensitivity (**d**) were tested by MTS assay. Downstream proteins of SNAI1 were examined by western blot (**e**). *P < 0.05 and **P < 0.01, compared with si-NC. **f**–**h** Indicated constructs (miR-Con, miR-30a, and/or SNAI1) were transfected in SW1990 cells. Cell growth (**f**) and gemcitabine sensitivity (**g**) were tested by MTS assay. Downstream proteins of SNAI1 were examined by western blot (**h**). *P < 0.05 and **P < 0.01, compared with miR-Con. ^#^*P* < 0.05 and ^##^*P* < 0.01, compared with miR-30a. Points, mean values for three independent experiments; error bars, ±SEM

Bioinformatics prediction indicated that IRS1, SNAI1, and SIRT1 are the potential target genes of miR-30a. As shown in Fig. 4a and Supplementary Figure 3A, miR-30a overexpression reduced IRS1 and SNAI1 protein level, while inhibition of miR-30a enhanced their expressions in SW1990 cells and Capan-2 cells. But miR-30a had no effect on SIRT1 expression, which is another predicted target gene of miR-30a (Supplementary Fig. 3B). These results indicated that SNAI1 and IRS1 might be the direct targets of miR-30a.

To further assess whether SNAI1 and IRS1 are the direct targets of miR-30a, we performed the luciferase reporter assay. As shown in Fig. 4b, overexpression of miR-30a diminished the reporter activity of wild-type SNAI1 gene, but had no significant effect on mutant SNAI1 gene where miR-30a binding site was mutated. On the other hand, miR-30a did not affect the luciferase reporter activity of IRS1 (Supplementary Fig. 3C). The above results clearly indicate that miR-30a specifically target SNAI1 at 3′UTR region by binding its putative sequence (Fig. 4b).

We also examined the direct effect of SNAI1 ablation on pancreatic cancer cell response to gemcitabine. Interestingly, SNAI1 ablation inhibited cell proliferation and increased the cell sensitivity to gemcitabine in SW1990 cells (Fig. 4c, d). The results were consistent with miR-30a effect. In addition, co-transfection of SNAI1 along with miR-30a partly reversed the effects of miR-30a alone (Fig. 4f, g). IRS1 also regulates pancreatic cancer cell growth and gemcitabine response (Supplementary Fig. 3D–E). Previous studies showed that SNAI1 repress SOCS3 expression via hypermethylation of the SOCS3 promoter^27^, and this further induces degradation of IRS1^28^. We also found that miR-30a regulated SNAI1, SOCS3 and IRS1 protein level (Supplementary Figure 3A–B, Fig. 4a, and h). Thus, perhaps IRS1 is indirectly regulated by miR-30a through SNAI1. Consistent with this hypothesis, knockdown of SNAI1 reduced IRS1 expression in SW1990 cells (Fig. 4e). Similarly, SNAI1 overexpression increased IRS1 expression (Fig. 4h). Moreover, as AKT and ERK1/2 are downstream effectors of IRS1^29,30^, and both have been shown to not only participate in complex biological processes but also contribute to chemoresistance and cancer progression^31–33^. Interestingly, knockdown of SNAI1 inhibited AKT and ERK phosphorylation, but had no effect on their total levels (Fig. 4e). MiR-30a overexpression also showed similar effect (Fig. 4h). The co-expression of SNAI1 and miR-30a reversed the miR-30a-mediated dephosphorylation of AKT and ERK (Fig. 4h). Thus, miR-30a functions are partially dependent on SNAI1/IRS1/AKT/ERK signaling pathway.

### Reduced miR-30a expression correlates with tumor progression and lower patient survival

**Fig. 5:**
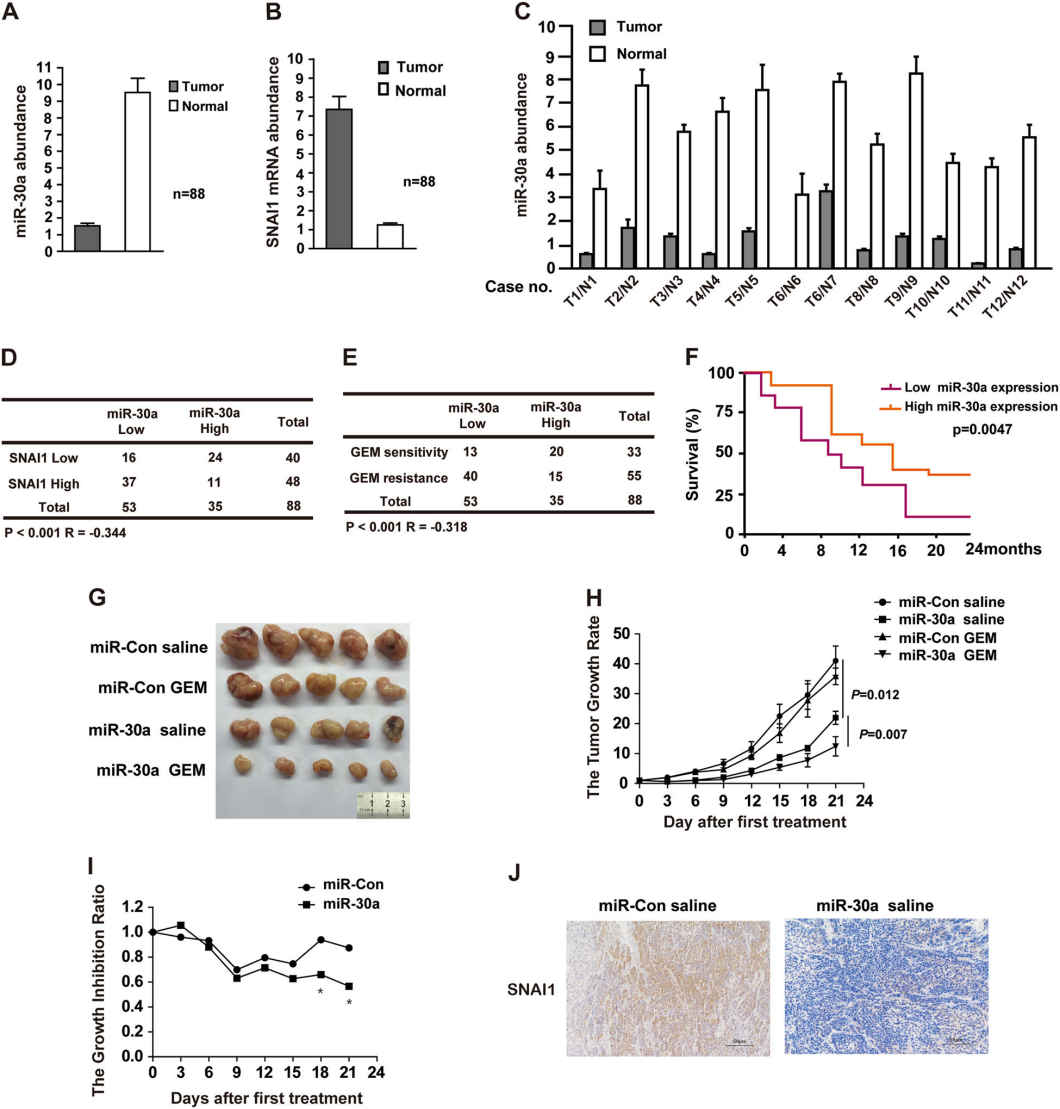
Reduced miR-30a expression correlates with tumor progression and lower patient survival rate in pancreatic cancers. **a** MiR-30a expression in tumor and adjacent normal pancreatic tissues was determined using qRT-PCR (88 pancreatic cancer samples and 88 normal pancreatic tissues). Error bars,  ±SEM. **b** SNAI1 gene expression in tumor and adjacent normal pancreatic tissues was determined using qRT-PCR (88 pancreatic cancer samples and 88 normal pancreatic tissues, as shown in **a**). Error bars,  ±SEM. **c** Representative miR-30a expression in tumor and normal pancreatic tissues was determined using qRT-PCR (12 pancreatic cancer samples and 12 normal pancreatic tissues). Error bars,  ±SEM. **d** Correlation study of miR-30a and SNAI1 in pancreatic carcinoma. Statistical analyses were performed with the *χ*^2^ test. *R*, Pearson correlation coefficient. **e** Correlation study between miR-30a level and gemcitabine response in pancreatic carcinoma with the *χ*^2^ test. *R*, Pearson correlation coefficient. **f** Association between miR-30a expression level and overall survival rate analyzed by the Kaplan–Meier method. Patients were split into high and low expression groups based on the median expression of the miR-30a. **g** Evaluation of tumor growth for xenograft mouse models of pancreatic cancer. Mice were inoculated subcutaneously in the right flank with 0.1 ml matrigel containing 2 × 10^6^ cells. Representative images of tumor were captured at the end of 3 weeks. *n* = 5 per group. **h** Gemcitabine response in miR-Con or miR-30a xenograft mice. Results are represented by the tumor volume measured at each time point normalized to day 0. Data are presented as means ± SEM (*n* = 5 per group). **i** MiR-30a xenografts are sensitive to gemcitabine in vivo. Results are represented by tumor inhibition ratio, calculated as follows: tumor volume at each time point normalized to day 0 for gemcitabine treated mice, corrected for that of vehicle group mice. *P < 0.05, compared with miR-Con. **j** SNAI1 expression was detected by IHC using xenograft tumor section. Points, mean values for three independent experiments; error bars, ±SEM

Since miR-30a sensitizes pancreatic cancer cells to chemotherapy and regulate cell proliferation, perhaps it is also involved in tumorigenesis. To confirm it, we compared the expression levels of miR-30a and SNAI1 in pancreatic cancer patient tissue and normal tissues samples. The clinicopathological features of patients were shown in Supplementary Fig. 4. And all the patients were treated with gemcitabine-based chemotherapy. As observed in Fig. 5a and c, the miR-30a expression was significantly lower in pancreatic tumor tissue samples in comparison to normal pancreatic tissue. Consistent with it, cancer tissue samples showed higher mRNA levels of SNAI1 (Fig. 5b). Moreover, as shown in Fig. 5d, a significantly negative correlation between miR-30a and SNAI1 mRNA levels (*P* < 0.001; *R* = −0.344) was observed in 88 pancreatic carcinomas. We also observed a strong negative correlation between miR-30a level and gemcitabine resistance (*P* < 0.001), because 75% (40 of 53) of miR-30a low samples showed gemcitabine resistance (Fig. 5e). These results indicate that miR-30a could serve as a marker for patient response to gemcitabine treatment and miR-30a-SNAI1 axis might have functional consequences in pancreatic tumorigenesis. To further test this possibility, we evaluated the role of miR-30a on tumor growth in vivo. Importantly, patients with higher miR-30a levels showed better 2-year survival rate (Fig. 5f). Furthermore, xenograft tumor growth experiment indicated that miR-30a significantly inhibited tumor growth in nude mice, and inclusion of gemcitabine had synergistic effect on tumor growth inhibition (Fig. 5g, h). The xenograft tumor section analysis confirmed reduced SNAI1 expression in samples from miR-30a injected nude mice (Fig. 5j). All of the above results imply that miR-30a-SNAI1 axis might contribute to tumor initiation. And miR-30a can serve as a new prognostic marker for human pancreatic cancers. Moreover, miR-30a also appeared to enhance gemcitabine response in vivo (Fig. 5e, g–i), thereby implicating miR-30a-SNAI1 axis as potential treatment option for pancreatic cancers.

### AKT-FOXO3a regulates miR-30a expression through a feedback loop

**Fig. 6:**
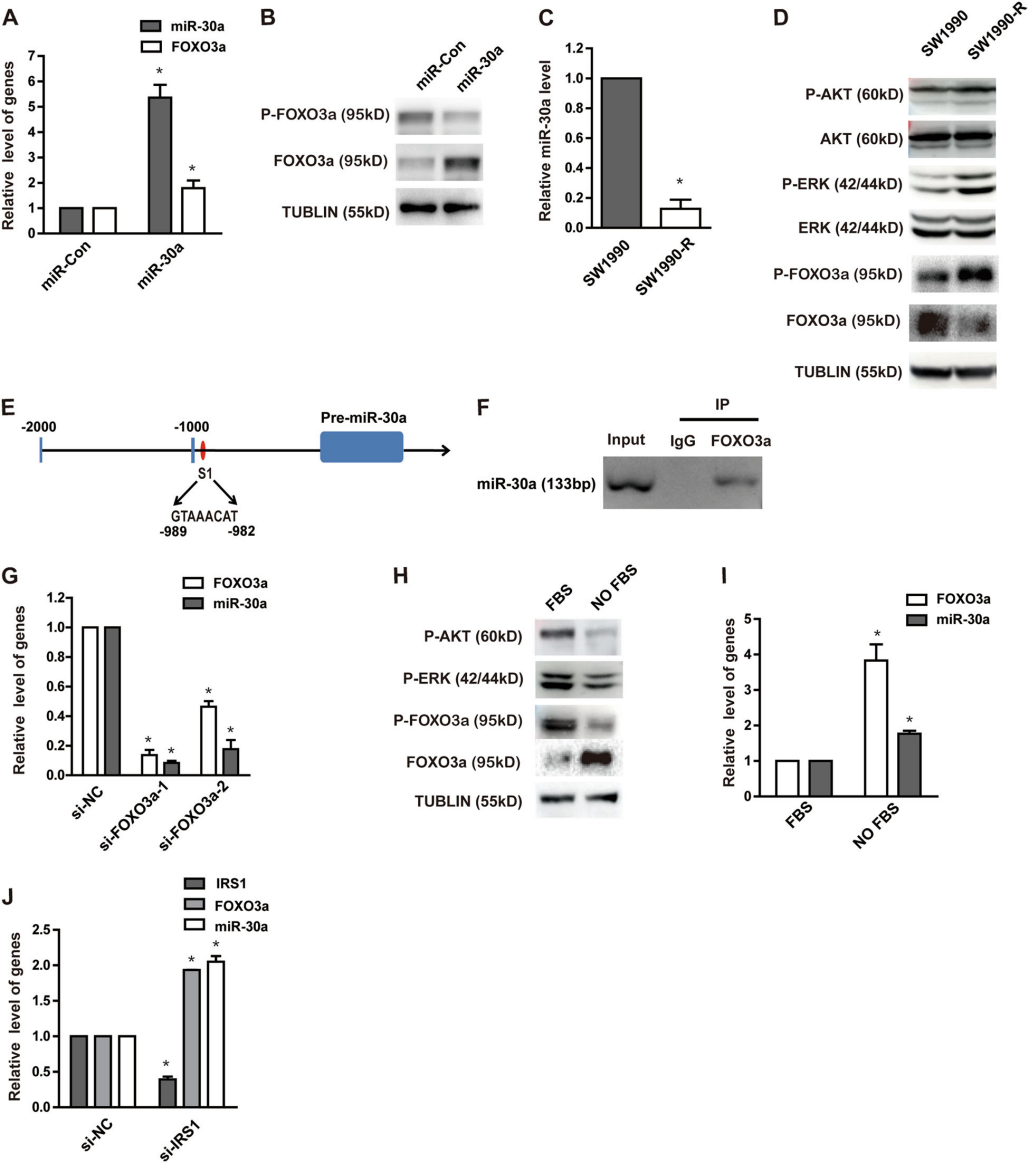
miR-30a and FOXO3a positively regulate each other to synergistically work as tumor suppressor. **a**, **b** SW1990 cells were transfected with indicated constructs. FOXO3a expression in SW1990 cells with or without miR-30a overexpression was detected by qRT-PCR (**a**). *P < 0.05, compared with control conditions. Protein expression of phospho-FOXO3a and total FOXO3a level were detected by western blot (**b**). **c** MiR-30a expression in SW1990-R cells and its parental cells was detected by qRT-PCR. *P < 0.05, compared with SW1990. **d** Gemcitabine-induced changes in protein expression between SW1990 cells and its parental cells were examined by western blot. **e** Computational prediction of FOXO3a binding site (S1) on 5’ miR-30a regulatory region. **f** Representative anti-FoxO3a ChIP-PCR showing FoxO3a binding to the miR-30a promoter. **g** Knockdown of FOXO3a with two different FOXO3a siRNAs (si-FOXO3a-1, si-FOXO3a-2) in SW1990 cells. MiR-30a level was determined by qRT-PCR subsequently. *P < 0.05, compared with control conditions. **h**, **i** SW1990 cells were treated with or without 10% FBS after overnight serum starvation. The P-AKT, P-ERK, P-FOXO3a, and FOXO3a in cell lysates were tested by western blot (**h**); mRNA level of FOXO3a and miR-30a were detected by qRT-PCR (**i**). *P < 0.05, compared with FBS group. **j** Transient knockdown of IRS1 with siRNA in SW1990 cells. MiR-30a and FOXO3a level were detected by qRT-PCR subsequently. *P < 0.05, compared with control conditions. Points, mean values for three independent experiments; error bars,  ±SEM

Finally, we sought to understand the mechanism of miR-30a downregulation in gemcitabine-resistance cells. Previous studies have revealed the importance of forkhead box O (FOXO) transcription factor family (such as FOXO3a), in mediating the downstream effects of insulin and AKT^34^. More specifically, FOXO3a is phosphorylated by AKT and ERK, which then is exported out of the nucleus and thereby results in suppression of FOXO3a transcriptional activity^35,36^. The FOXO3a phosphorylation by ERK was also shown to reduce FOXO3a via MDM2-mediated proteasome degradation^37^. As we observed inhibition of AKT and ERK pathway in miR-30a upregulated cells, it was obvious to hypothesize that there might be some correlation between FOXO3a levels and miR-30a expression. Indeed, miR-30a overexpression resulted in upregulation of total FOXO3a protein levels and downregulation of phosphorylated FOXO3a levels, due to reduced phospho-AKT and phospho-ERK levels in these cells (Fig. 6a, b). Moreover, phosphorylated FOXO3a was increased, while total FOXO3a was decreased in SW1990-R cells after reduced miR-30a expression (Fig. 6c, d). Next, we also analyzed if FOXO3a has any effect on miR-30a transcriptional regulation. The miR-30a upstream region (−1 to −2000) was analyzed using JASPAR (http://jaspar.genereg.net) software to identify any FOXO3a binding site. We found one potential binding sites of FOXO3a on the upstream region of miR-30a (site 1) (Fig. 6e). ChIP assay also showed that FOXO3a binds the upstream region of miR-30a gene (Fig. 6f). In parallel, we also ablated FOXO3a by its siRNA in SW1990 cells, and observed that reduced FOXO3a expression markedly decreased miR-30a levels, as shown in Fig. 6g. To independently verify this observation, we activated AKT and ERK in SW1990 cells by fetal bovine serum (FBS). In comparison to FBS treated cells, the non-treated cells showed dramatically decreased FOXO3a phosphorylation, and elevated total FOXO3a and miR-30a levels (Fig. 6h, i). Similarly, reduced IRS1 levels, which phosphorylates both AKT and ERK, also led to the elevation of FOXO3a and miR-30a levels (Fig. 6j), which further demonstrated the positive effect of FOXO3a on miR-30a expression. Taken together, our results confirmed the existence of a feedback loop, where AKT-FOXO3a and miR-30a directly regulates each other.

## Discussion

**Fig. 7:**
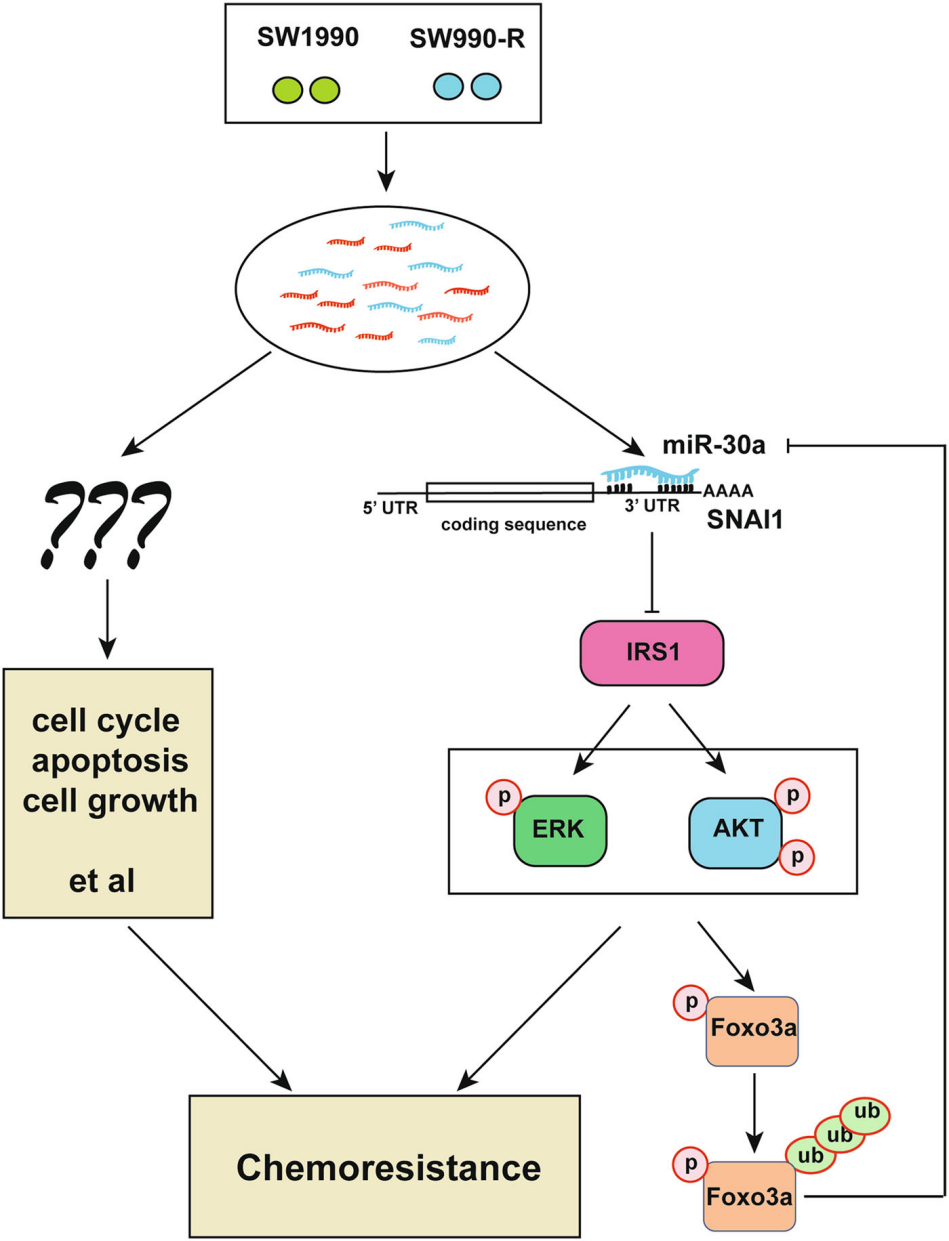
Working model of miRNAs functions in chemoresistance. MiR-30a regulates cancer cell response to chemotherapy through SNAI1/IRS1/ERK/AKT pathway. On the other hand, miR-30a is a downstream target gene of AKT-FOXO30a that forms a feedback loop. In addition, it is highly possible that other miRNAs identified by miRNA screen may also be involved in gemcitabine response and are worth exploring further

Development of resistance to gemcitabine usually represents a major challenge for pancreatic cancer treatment. Therefore, identification of specific biomarkers for chemoresistance, and understanding the underlying mechanism will provide a way forward to develop possible therapeutic strategies to overcome this problem. Previous studies have identified multiple oncogenes or tumor suppressor genes as biomarkers for gemcitabine response^25,38,39^. However, the involvement of non-coding RNAs, like miRNAs, in gemcitabine response has not been very clear. It is well known that the microRNAs (miRNAs) likely regulate most biological processes and can be employed to better understand the complex processes, including drug resistance in cancer. In this context, the miRNA expression profiling can provide a useful tool to identify miRNAs that probably have an important role in various aspects of cancer, including specific drug response. Therefore, we delineated a genome-wide expression profile of miRNA and identified key pathways associated with gemcitabine response in pancreatic cancer. We found that miRNAs dysregulation, especially miR-30a downregulation, probably results in developing gemcitabine resistance in pancreatic cancer. Further mechanistic studies revealed that miR-30a targets SNAI1-AKT pathway, which modulated AKT/ERK phosphorylation and activity, and thereby controlling the cell survival upon gemcitabine treatment. On the other hand, we also found that AKT-FOXO3a-miR-30a forms a positive feedback loop to suppress the pancreatic tumor progression (Fig. 7).

Since AKT/ERK signaling pathway has been a major signaling node within the cells^40–42^, its activity needs to be tightly regulated. Dysregulation of this pathway can disrupt the balance between cell survival and death, and can impact cancer development and therapy. Indeed, the PI3K-AKT pathway has been linked with the development of chemotherapeutic resistance in a variety of drugs, including gemcitabine^43^, etoposide^44^, and taxol^45^. In addition, the hyper-phosphorylation of this pathway has also been linked with poor prognosis in variety of cancers^46,47^. Among the SNAI1/IRS1/AKT/ERK pathway, SNAI1 is one of the main members of the Snail family of transcription factors, which has been shown to be an important mediator of epithelial–mesenchymal transition^48^ and is involved in tumor progression^49^. IRS1 is a signaling adapter protein, which integrates different signaling cascades. IRS1 protein has already been implicated in various cancer, including colorectal^50^, lung^51^, prostate^52^, and breast cancer^53^. In our study, we also noticed that dysregulation of miR-30a along with SNAI1 was associated with poor prognosis in pancreatic cancer.

Many IRS1/ERK/AKT pathway regulators like IRS1, PI3K, PTEN, and AKT are typically mutated or dysregulated in cancer, so it is plausible that miR-30a downregulation may also contribute to not only chemoresistance but also tumorigenesis. We noticed lower miR-30a expression in pancreatic cancer, supporting a potential tumor suppressor role of miR-30a. Interestingly, we also observed the crosstalk between FOXO3a and miR-30a, and FOXO3a has already been shown to have an important role as tumor suppressor, through transcriptionally regulating cell cycle- and apoptosis-associated genes such as p27, cyclin D1, and TRAIL^54–58^. Thus, we believe that feedback loop will amplify the anti-tumor effects of both FOXO3a and miR-30a.

Overall, our study identified important miRNAs involved in gemcitabine resistance in pancreatic cancer cells. Furthermore, since dysregulation of IRS1/ERK/AKT pathways is frequently linked to cancer predisposition and poor prognosis, our findings may also have important implications for pancreatic cancer etiology and chemotherapy.

## Materials and methods

### Generation of SW1990-R cells

SW1990 cells were plated at 70% confluence in 10-cm dishes with RPMI 1640 containing 10% FBS. Cells were cultured in medium with gemcitabine starting from 50 nM for 72 h. Cells were passaged when they reached 100% confluence. The concentration of gemcitabine was increased in half-log intervals per two passages, until a final concentration of 4 μM was achieved. The resistance cells (designated as SW1990-R) were maintained in RPMI 1640 with 10% FBS and 4 μM gemcitabine.

### Small RNA sequencing and data analysis

Total RNA from SW1990 and SW1990-R cells were extracted using TRIzol reagent (Invitrogen, Carlsbad, CA, USA), and their quantity and purity were measured using NanoPhotometer® spectrophotometer (IMPLEN, CA, USA). RNA integrity was assessed using RNA Nano 6000 Assay Kit on Agilent Bioanalyzer 2100 system (Agilent Technologies, CA, USA). Three micrograms of the total RNA sample was used as input material for the creation of small RNA libraries. Total of four sequencing libraries were generated using NEBNext® Multiplex Small RNA Library Prep Set from Illumina® (NEB, USA) following manufacturer’s recommendations. The quality of the libraries was assessed with Agilent Bioanalyzer 2100 system using DNA High Sensitivity Chips. Finally, the library preparations were sequenced on an Illumina HiSeq 2500/2000 platform to generate 50 bp single-end reads.

Next the raw sequencing data was screened to filter low quality reads, including reads lacking 3′-adaptors, 5′-adaptor contaminants and containing polyA/T/G/C. The clean reads of each sample were screened for sRNAs of 18–35 bp for subsequent analysis, by mapping them to human genome (release hg19) by Bowtie^59^ to analyze their expression and distribution. Subsequently, the mapped reads were compared to the miRBase (20.0) to annotate conserved miRNAs. MiREvo and miRDeep2 analysis platforms were used to predict novel miRNAs.

The potential miRNAs targets were predicted using miRanda software. In addition, the GOSeq/topGO release 2.12 method was used for gene ontology enrichment assay, while KOBAS v2.0 software was used to identify KEGG pathway terms enriched in the predicted target genes.

### Cell lines and drugs

Human pancreatic cancer cell line PANC-1, SW1990, and BxPC-3 were obtained from China Infrastructure of Cell Line Resources (Beijing, China). Capan-2 and MIA PaCa-2 were purchased from Obio Technology (Shanghai, China) Corp., Ltd. SW1990, PANC-1 and MIA PaCa-2 were cultured in DMEM, Capan-2 was maintained in McCoy’s 5A and BxPC-3 was cultured in RMPI 1640, all supplemented with 10% fetal bovine serum, 100 U/ml of penicillin and 100 μg/ml of streptomycin. All of the cells were incubated at 37 ℃ in a humidified atmosphere of 5% CO_2_. The gemcitabine (Gemzar) drug was purchased from Eli Lilly and Company (Indianapolis, Indiana, USA).

### Plasmids, siRNAs, and transfection

The lentivirus containing miRNA precursor, oligodeoxyribonucleotide or negative control were purchased from Obio Technology (Shanghai, China) Corp., Ltd.

The pEnter-SNAI1 plasmid was obtained from ViGene BioSciences (Shandong, China), Inc. All cell transfections were performed using Lipofectamine® 2000 transfection reagent (Invitrogen, Carlsbad, CA, USA) according to the manufacturer’s recommendations. The siRNAs targeting SNAI1, IRS1 and FOXO3a, along with negative control, were synthesized by Shanghai GenePharma Co., Ltd and were transfected at 100 nM concentration using Lipofectamine™ RNAiMAX reagent (Invitrogen, Carlsbad, CA, USA) according to the manufacturer’s instructions.

The following two siRNA sequences (sense strands) were used to target IRS1; (1) GGUCAGACAAAGAACCUGATT, and (2) GAGGAGCUAAGCAACUAUATT. The two siRNA sequences (sense strands) targeting FOXO3a were: (1) CAAGCACAGAGUUGGAUGATT, and (2) CGUGAUGCUUCGCAAUGAUTT. The two siRNA sequences (sense strands) targeting SNAI1 were: (1) GCUUUGAGCUACAGGACAATT, and (2) GCUGCAGGACUCUAAUCCATT

### MTS assay

Cell proliferation and gemcitabine response were determined by using CellTiter 96 Aqueous One Solution Cell Proliferation Assay (Promega, Madison, WI, USA). A total of 3 × 10^3^ cells per well were seeded into 96-well plates in triplicates for drug response. The plated cells were treated with 10-fold diluted concentrations of gemcitabine from 0.001 to 1000 μM for 72 h. Later the plates were analyzed in a SpectraMax 190 plate reader (Molecular Devices, Sunnyvale, CA, USA) at a wavelength of 490 nm. For cell proliferation assay, 1 × 10^3^ cells per well were seeded into 96-well plate in triplicates. And the plates were read as described above, at indicated time points.

### Colony formation assay

Total of 800 cells were seeded in a 3.5 cm dish, in triplicate, and cultured in complete medium for 2 weeks. Colonies were then fixed with anhydrous methanol and stained with crystal violet (0.1% w/v) dye. The counting of the colonies was performed using Image J software. Colony forming efficiency was calculated as the percentage of colonies to the number of plated cells.

### Quantitative real-time reverse transcription PCR (qRT-PCR)

Total RNA was isolated using Trizol Reagent (Invitrogen, Carlsbad, CA, USA). For mRNA analysis, 1 μg of total RNA was reverse transcribed using FastQuant RT Kit (TIANGEN, Beijing, China). Next, the real-time PCR was performed using Power SYBR Green PCR master mix (Toyobo, Osaka, Japan), and data were normalized to β-actin expression. The All-in-One™ miRNA qRT-PCR Detection Kit (GeneCopoeia, Rockville, MD, USA) was used to quantitatively measure miRNAs, according to the manufacturer’s protocol, and the relative amount of miRNAs was normalized against U6. All these experiments in triplicate were performed on a Bio-Rad CFX96 machine (Bio-Rad, Hercules, CA, USA), and the fold changes for both miRNA and mRNA were calculated by 2^−△△CT^ method. The primers for miRNA were purchased from Fulengen (Guangzhou, China). The primer sequences used for mRNA detection are listed as follows: IRS1 forward: ACTGGACATCACAGCAGAATGA; and IRS1 reverse: AGAACGTGCAGTTCAGTCAA. FOXO3a forward TGGTTTGAACGTGGGGAACT; and FOXO3a reverse: CAGTTTGAGGGTCTGCTTTGC.

### Western blotting

After harvesting, the cells were lysed on ice using NETN buffer (20 mM Tris–HCl, pH 8.0, 100 mM NaCl, 1 mM EDTA, 0.5% Nonidet P-40) containing 10 mM NaF and 1 mM PMSF. Proteins were separated by 10% SDS-PAGE, and transferred to PVDF membrane, to be probed with corresponding primary antibodies at 4 ℃, overnight. Next day, the membranes were incubated with corresponding secondary antibodies, conjugated with horseradish peroxidase, for 1 h, and then ECL detection reagent was used to visualize the target proteins. Primary antibodies against AKT (#9272, 1:1000), phospho-AKT (Ser473) (#9271, 1:1000), phospho-FOXO3a (Thr32) (#9464, 1:1000), and ERK (#9102, 1:1000) were all purchased from Cell Signaling Inc. (Boston, MA, USA). IRS1 (#559, 1:1000), SOCS3 (#73045, 1:200), SIRT1 (#74504, 1:200), SNAI1 (#271977, 1:200), and phospho-ERK (E-4, 1:1000) were obtained from Santa Cruz Biotechnology (Santa Cruz, CA, USA).

### Luciferase assays

PmirGLO Dual‐Luciferase miRNA Target Expression Vector containing with wild-type or mutant 3′‐UTR of SNAI1/IRS1 were obtained from shanghai GenePharma Co., Ltd. 293T cells plated into a 24-well plate were co-transfected with 100 nM of either miR-30a mimic or negative control oligo and 200 ng reporter comprising wild-type or mutant 3′‐UTR. The luciferase activity was measured by Dual‐Luciferase Reporter Assay System (Promega, USA) 24 h after transfection.

### Chromatin immunoprecipitation (ChIP) analysis

SW1990 cells cultured in 10 cm dishes were cross-linked with 1% formaldehyde for 10 min at 37 ℃. Cross-linked cells were then lysed and subjected for ChIP assay using FOXO3a antibody (ab12162, Abcam, Cambridge, UK) with One-Day Chromatin Immunoprecipitation Kit (Millipore) according to the manufacturer’s instructions. Later PCR analysis of the purified DNA was performed using following miR-30a primers: Forward: 5′-ACCCAACAGAAGGCTAAAGAAG-3′; Reverse: 5′-TTGAAGTCCGAGGCAGTAGG-3′

### Xenograft tumor study

4-week-old-female BALB/c athymic nu/nu mice were purchased from SiBeiFu (Beijing, China), and were maintained in animal breeding facility of National center for protein science. The SW1990 cells (2 × 10^6^) stably infected with miR-Con or miR-30a-overexpressing lentivirus were injected into the right flank of each mice using 0.1 ml matrigel. After the xenograft tumor volumes reached 100 mm^3^, the mice injected with miR-30a overexpressing cells were randomly divided into two groups (*n* = 5 per group). The one group was injected with physiological saline, every 3 days for 7 times, while other group received drug gemcitabine at a dose of 50 mg/kg, every 3 days for 7 times. Also, the mice injected with miR-Control cells were also grouped as described above and were similarly treated. The mice weight and tumor volume were measured twice a week from day 0 to 21, where day 0 represented the day of initial treatment and day 21 was the day of killing. All tumor volumes were calculated by using the formula *V* = *L* × *W*^2^ × *π*/6 (*V*, volume; *L*, long diameter; *W*, short diameter of tumor).

### Statistical analysis

All experimental data were expressed as mean ± SEM, and the statistical analyses were performed using Student’s *t*-test, ANOVA or *χ*^2^ test. Spearman correlation coefficients were calculated to estimate the correlations. *P*-value of <0.05 represented statistically significant difference.

## Supplementary information

Supplementary information

Supplementary Figure1

Supplementary Figure2

Supplementary Figure3

Supplementary Figure4
