# Supplementary material for: MiR-30a regulates cancer cell response to chemotherapy through SNAI1/IRS1/AKT pathway
Source: Cell Death Dis. 2019 Feb 15;10(3):153. doi: 10.1038/s41419-019-1326-6 (PMC6377638; doi:10.1038/s41419-019-1326-6)
Supplement: Supplementary file 1 — Supplementary information [file 41419_2019_1326_MOESM1_ESM.docx]

**SUPPLEMENTARY FIGURELEGENDS**

**Supplementary Figure1: Growth inhibition by gemcitabine in five different pancreatic cancer cell lines.**

**(A)** Cell survival was detected by MTS assay after treatment with gemcitabine for 72 hrs.

**(B)** The 50% inhibitory concentration (IC50) of five pancreatic cancer cell lines was calculated. Points, mean values for three independent experiments; Error bars, +/− SEM.

**(C)** The expression levels of miR-30a in cell lines Capan-2, BxPC-3, SW1990, MIA-PaCa-2 and PANC-1 were detected by qRT-PCR. The relationship between the expression level of miR-30a in each cell and their corresponding IC50 to gemcitabine were shown.

**Supplementary Figure2：Ectopic expression of miR-30a suppresses cell proliferation and enhances cell sensitivity to gemcitabine in Capan-2 cells.**

**(A-B)** Capan-2 cells were transfected with miR-30a or miR-Con. MiR-30a level was confirmed by qRT-PCR **(A)**. Gemcitabine sensitivity was determined by MTS assays **(B)**. **P*<0.05 and ***P*<0.01, compared with miR-Con.

**(C-D)** Capan-2 cells were transfected with miR-30a inhibitor or miR-Con. MiR-30a expression was detected by qRT-PCR **(C)**. Gemcitabine sensitivity was determined by MTS assays **(D)**. **P*<0.05 and ***P*<0.01, compared with miR-Con.

**(E)** Capan-2 cells were transfected with indicated constructs and then treated with 2uM gemcitabine for 72 hrs. The colony forming assay was performed. Representative micrographs (left) and quantification (right) of crystal violet-stained cell colonies were displayed.

**(F)** Capan-2 cells were transfected with indicated constructs. Cells were then treated and detected as in **(E).**

**(G-H)** Capan-2 cells were transfected with miR-30a or miR-Con. Cell growth were determined by MTS assays **(G)**. Representative micrographs (left) of colony formation, as well as quantification (right) of crystal violet-stained cell colonies were displayed **(H)**. n=3 wells per group. **P*<0.05 and ***P*<0.01, compared with miR-Con.

**(I-J)** Capan-2 cells were transfected with miR-30a inhibitor or miR-Con. Cell growth and colony formation assay were determined as in **(G) and (H)**, respectively. n=3 wells per group. **P*<0.05 and ***P*<0.01, compared with miR-Con.

Points, mean values for three independent experiments; Error bars, +/− SEM.

**Supplementary Figure3:**

**(A)** Capan-2 cells were transfected with the indicated constructs, IRS1 and SNAI1 level were checked by western blot.

**(B)** The effect of miR-30a modulation (miR-con, miR-30a and miR-30a inhibitor) on SOCS3 and SIRT1 protein expression by western blot analysis in Capna-2 and SW1990 cells.

**(C)** The effect of miR-30a on IRS1 was tested by luciferase reporter assay. Seed sequences for miR-30a on the IRS1 3´UTR (left) and luciferase activities of 293T cells co-transfected with pmirGlo plasmids encoding wild-type/mutated 3’UTR sequences of IRS1 and oligos (right) were displayed. The data are represented as mean ± SEM. (n=3).

**(D-E)** Capan-2 cells were transfected with indicated siRNA. Cell growth **(D)** and gemcitabine sensitivity **(E)** were tested by MTS assay. **P*<0.05 and ***P*<0.01, compared with si-NC.

**Supplementary Figure4:** The clinicopathological features of 88 pancreatic cancer patients.
