# Supplementary figures and images for: MiR-30a regulates cancer cell response to chemotherapy through SNAI1/IRS1/AKT pathway
Source: Cell Death Dis. 2019 Feb 15;10(3):153. doi: 10.1038/s41419-019-1326-6 (PMC6377638; doi:10.1038/s41419-019-1326-6)

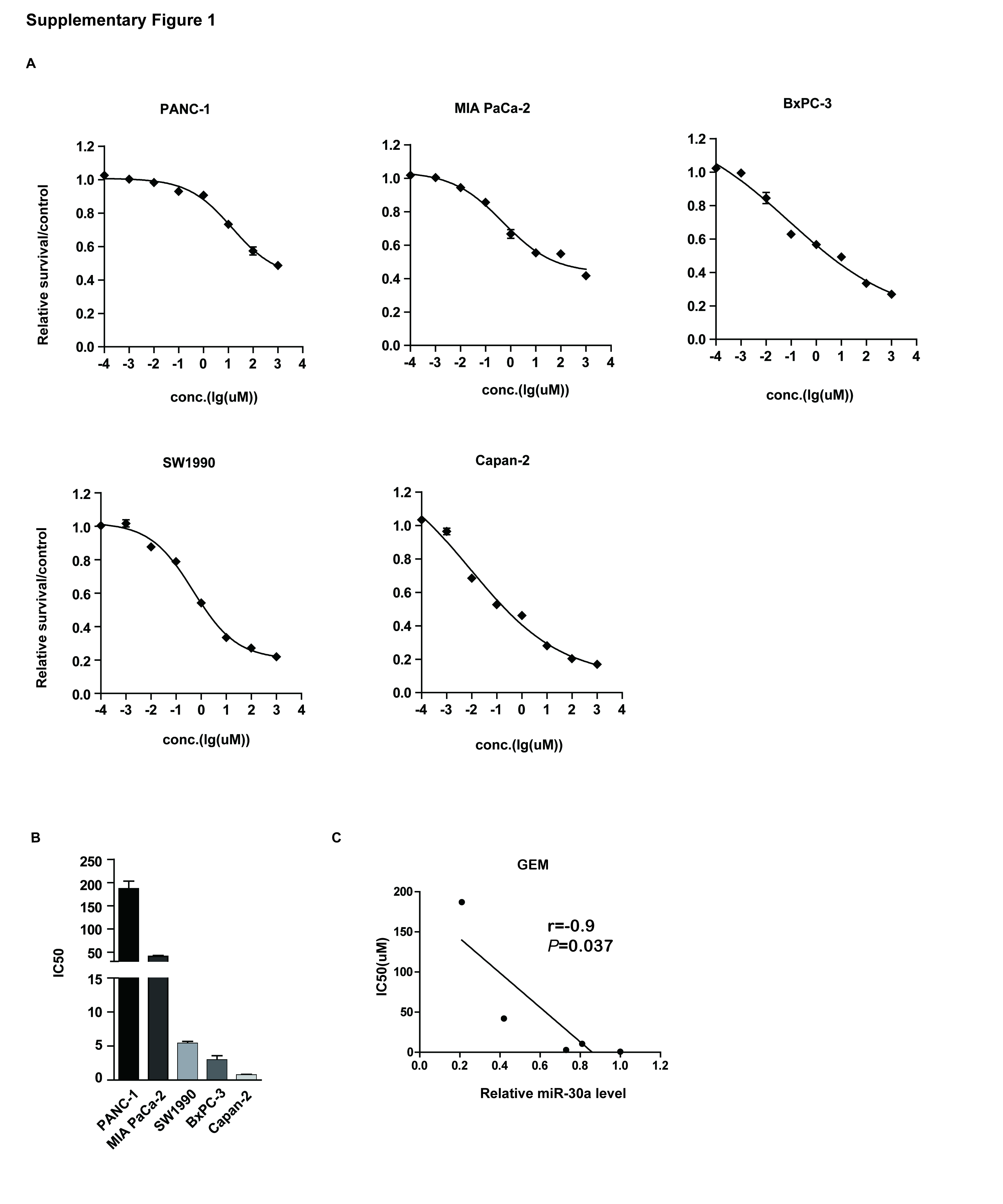

Supplement: Supplementary file 2 — Supplementary Figure1 [file 41419_2019_1326_MOESM2_ESM.tif]

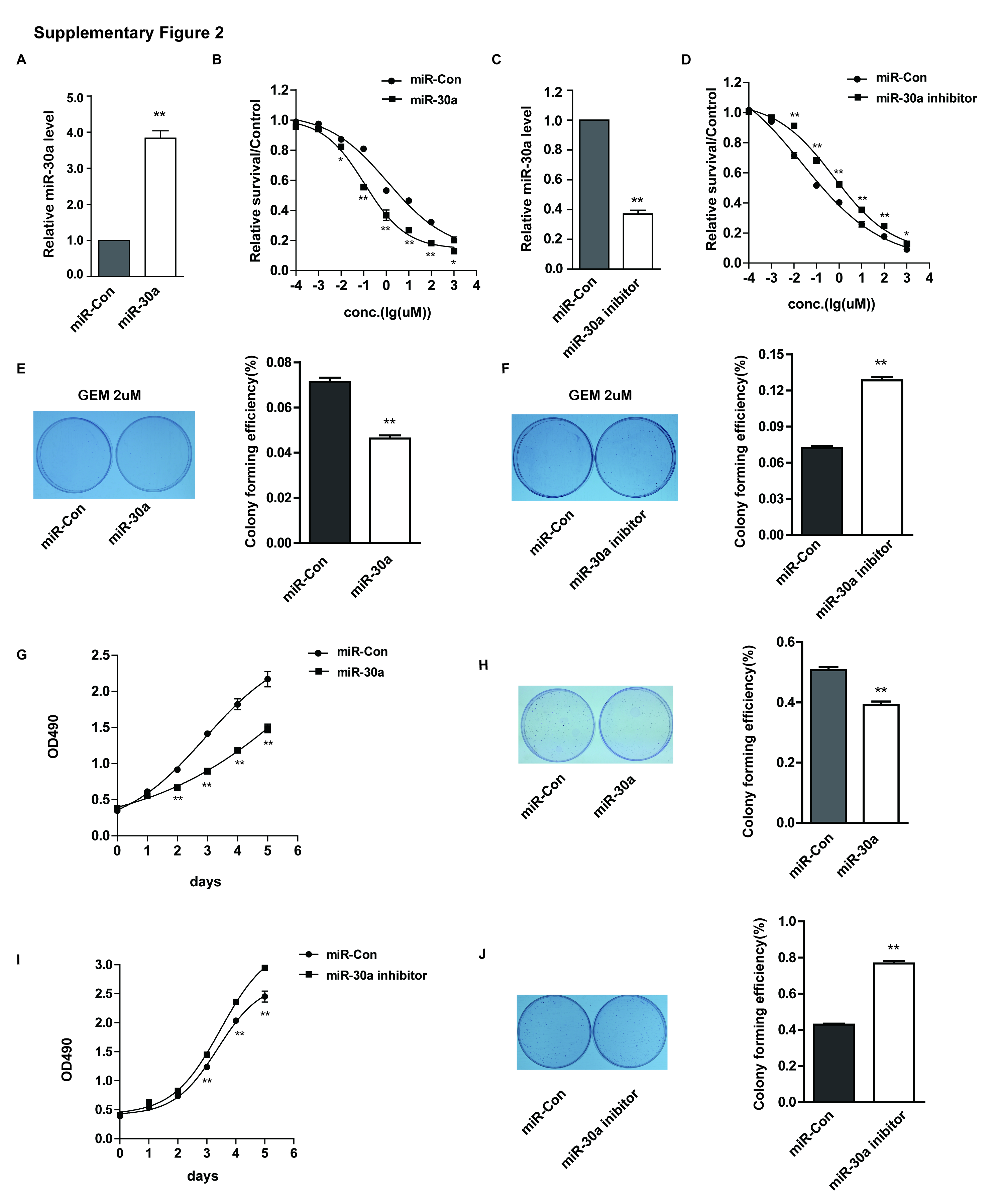

Supplement: Supplementary file 3 — Supplementary Figure2 [file 41419_2019_1326_MOESM3_ESM.tif]

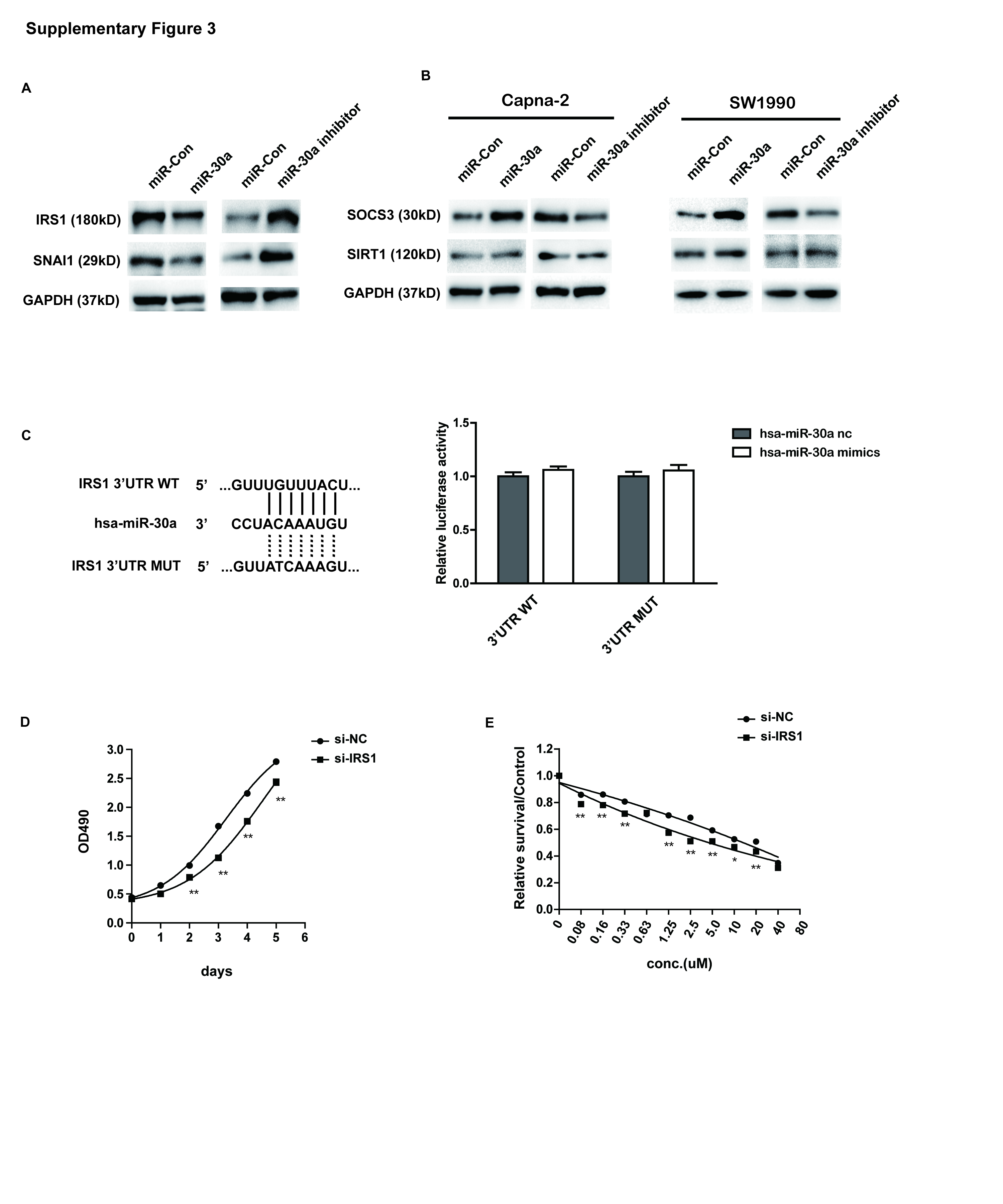

Supplement: Supplementary file 4 — Supplementary Figure3 [file 41419_2019_1326_MOESM4_ESM.tif]

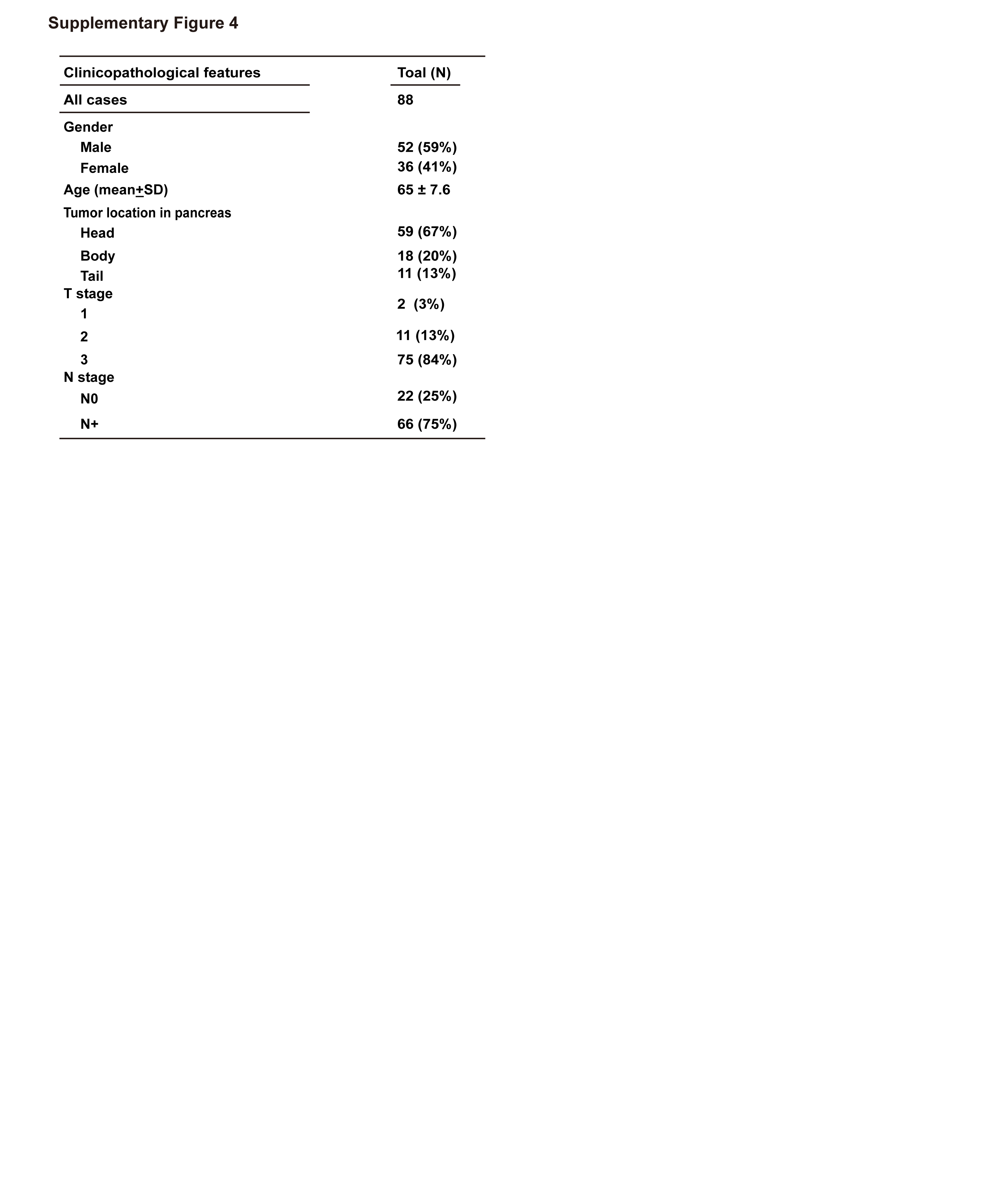

Supplement: Supplementary file 5 — Supplementary Figure4 [file 41419_2019_1326_MOESM5_ESM.tif]
